# Supplementary material for: Optimum Fermentation Conditions for Bovine Lactoferricin-Lactoferrampin-Encoding LimosiLactobacillus reuteri and Regulation of Intestinal Inflammation
Source: Foods. 2023 Nov 9;12(22):4068. doi: 10.3390/foods12224068 (PMC10670345; doi:10.3390/foods12224068)
Supplement: Supplementary file 1 [file foods-12-04068-s001.zip › foods-2680516-supplementary.pdf]

foods

MDPI

Supplementary Materials

Optimum Fermentation Conditions for Bovine Lactoferricin-Lactoferrampin-Encoding *LimosiLactobacillus reuteri* and Regulation of Intestinal Inflammation

Weichun Xie [1,†], Xueying Wang [1,†], Jiyao Cai [1], Huitao Bai [1], Yilan Shao [1], Zhuoran Li [1], Limeng Cai [1], Senhao Zhang [1], Jiaxuan Li [1], Wen Cui [1], Yanping Jiang [1,*] and Lijie Tang [1,2,*]

[1] College of Veterinary Medicine, Northeast Agricultural University, Harbin 150030, China; xieweichun_neau@163.com (W.X.); tycoon28644@163.com (X.W.); caijiyao0723@163.com (J.C.); bht17077876222@163.com (H.B.); shaoyilan@neau.edu.cn (Y.S.); lizhuoran1009@163.com (Z.L.); clmyoyo@163.com (L.C.); zhang_sen_hao@163.com (S.Z.); lijiaxuan.1993@163.com (J.L.); cuiwen@neau.edu.cn (W.C.)
[2] Heilongjiang Key Laboratory for Animal Disease Control and Pharmaceutical Development, Northeast Agricultural University, Harbin 150030, China
* Correspondence: jiangyanping@neau.edu.cn (Y.J.); tanglijie@163.com or tanglijie@neau.edu.cn (L.T.); Tel.: +86-451-5519-0385 (Y.J.); +86-451-5519-0824 (L.T.)
† These authors contributed equally to this work.

Citation: Xie, W.; Wang, X.; Cai, J.; Bai, H.; Shao, Y.; Li, Z.; Cai, L.; Zhang, S.; Li, J.; Cui, W.; et al. Optimum Fermentation Conditions for Bovine Lactoferricin-Lactoferrampin-Encoding *LimosiLactobacillus reuteri* and Regulation of Intestinal Inflamma-tion. *Foods* **2023**, *12*, 4068. https://doi.org/10.3390/foods12224068

Academic Editor: Xuetuan Wei

Received: 9 October 2023
Revised: 2 November 2023
Accepted: 4 November 2023
Published: 9 November 2023

Copyright: © 2023 by the authors. Licensee MDPI, Basel, Switzerland. This article is an open access article distributed under the terms and conditions of the Creative Commons Attribution (CC BY) license (https://creativecommons.org/licenses/by/4.0/).

Abstract: The multifunctional antibacterial peptide lactoferricin-lactoferrampin (LFCA) is derived from bovine lactoferrin. Optimization of the fermentation process should be studied since different microorganisms have their own favorable conditions and processes for growth and the production of metabolites. In this study, the culture conditions of a recombinant strain, pPG-LFCA-E/LR-CO21 (LR-LFCA), expressing LFCA was optimized, utilizing the high-density fermentation process to augment the biomass of *LimosiLactobacillus reuteri* and the expression of LFCA. Furthermore, an assessment of the protective effect of LR-LFCA on intestinal inflammation induced by lipopolysaccharide (LPS) was conducted to evaluate the impact of LR-LFCA on the disease resistance of piglets. The findings of this study indicate that LR-LFCA fermentation conditions optimally include 2% inoculation volume, 36.5 °C fermentation temperature, 9% dissolved oxygen concentration, 200 revolutions/minute stirring speed, pH 6, 10 mL/h glucose flow, and 50% glucose concentration. The inclusion of fermented LR-LFCA in the diet resulted in an elevation of immunoglobulin levels, significant upregulation of tight junction proteins ZO-1 and occludin, reinforcement of the intestinal barrier function, and significant amelioration of the aberrant alterations in blood physiological parameters induced by LPS. These results offer a theoretical framework for the implementation of this micro-ecological preparation in the field of piglet production to enhance intestinal well-being.

Keywords: lactoferricin-lactoferrampin (LFCA); *LimosiLactobacillus reuteri*; protein expression; high-density fermentation; intestinal inflammation

Table S1. Primer sequence of Real-time fluorescence quantitative PCR.

Gene	Primer sequence
GAPDH	F: 5'-TCATCATCTCTGCCCCTTCT-3'<br>R: 5'-GTCATGAGTCCCTCCACGAT-3'
TGF-β	F: 5'-GAAGCGCATCGAGGCCATTC-3'<br>R: 5'-GGCTCCGGTTCGACACTTTC-3'
IL-10	F: 5'-GACCAGATGGGCGACTTGTTG-3'<br>R: 5'-GGGAGTTCACGTGCTCCTTGAT-3'
TNF-α	F: 5'-GCCCTTCCACCAACGTTTTC-3'<br>R: 5'-TCCCAGGTAGATGGGTTCGT-3'
IL-6	F: 5'-CCTCGGCAAAATCTCTGCAA-3'<br>R: 5'-TGAAACTCCACAAGACCGGT-3'
IL-1β	F: 5'-GCCAGTCTACATTGCTCATGTTTCT-3'<br>R: 5'-GTTGTCACCATTGTTAGCCATCAC-3'
ZO-1	F: 5'-AGCCCGAGGCGTGTTT-3'<br>R: 5'-GGTGGGAGGATGCTGTTG-3'
Occludin	F: 5'-GCACCCAGCAACGACAT-3'<br>R: 5'-CATAGACAGAATCCGAATCAC-3'

Table S2. The regulatory effects of LR-LFCA on blood physicochemical indexes in LPS-treated piglets.

Items	Control	LPS	LR-LFCA	Reference range
RBC (10^{12}/L)	6.38±0.03	6.04±0.16	6.23±0.11	5.00~8.00
HCT (%)	37.7±0.02	35.35±0.45	36.07±0.27	32.0~50.0
HGB (g/dL)	14.43±0.99^{a}	10.46±0.83^{b}	13.36±0.75^{a}	10.7~16.7
MCV (fL)	58.38±1.55^{b}	67.22±0.02^{a}	55.96±1.45^{b}	50.0~68.0
MCHC (g/dL)	31.53±0.57^{b}	26.26±0.15^{a}	32.08±0.10^{b}	30.0~34.0
MCH (pg)	19.48±0.15^{c}	15.34±0.10^{a}	21.05±0.57^{b}	17.0~21.0
WBC (10^{9}/L)	19.21±0.51^{c}	38.91±0.48^{a}	23.95±0.04^{b}	11.00~22.00
NEU (10^{9}/L)	6.98±0.34^{a}	15.46±0.15^{b}	7.03±0.12^{a}	4.48~7.52
%NEU (%)	38.33±0.81^{a}	39.67±0.32^{a}	29.35±0.43^{b}	
LYM (10^{9}/L)	7.80±1.80^{a}	12.12±1.20^{b}	8.25±0.55^{a}	6.60~18.70
%LYM (%)	40.6±0.39^{a}	31.14±0.17^{b}	34.42±0.56^{c}	
MONO 10^{9}/L	0.95±0.14^{b}	1.53±0.18^{a}	0.81±0.04^{b}	0.3~1.25
PLT (K/μL)	459±5.88^{c}	554±2.63^{a}	502±6.11^{b}	300~700

Note: Data are represented as the mean ± SD. Different lowercase letters represent $P < 0.05$.

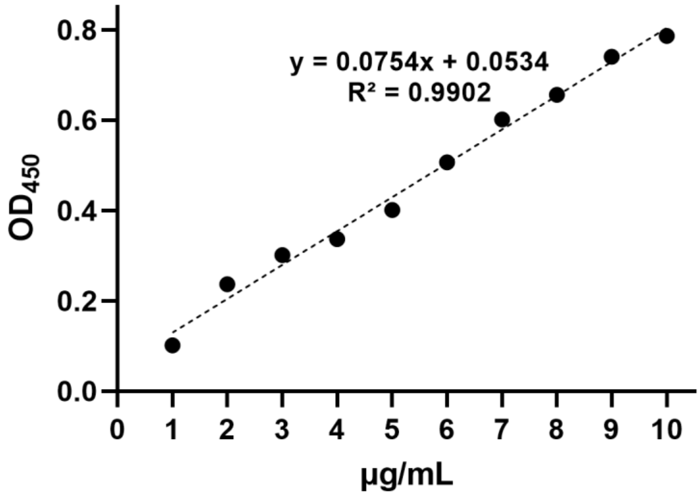

Figure S1. Established the quantity standard curve of LFCA by ELISA.

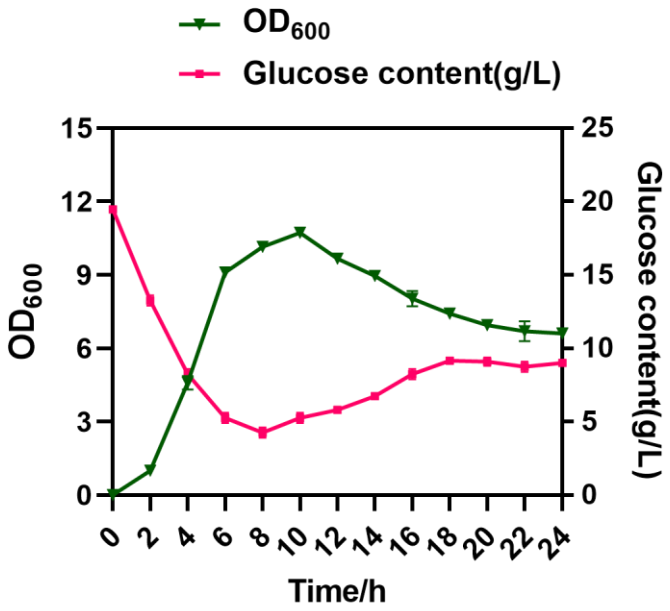

Figure S2. Growth and glucose consumption curve of LR-LFCA at pH 6. Four hours following fermentation, 50% (w/w) glucose was supplemented at a flow rate of 10 mL/h.

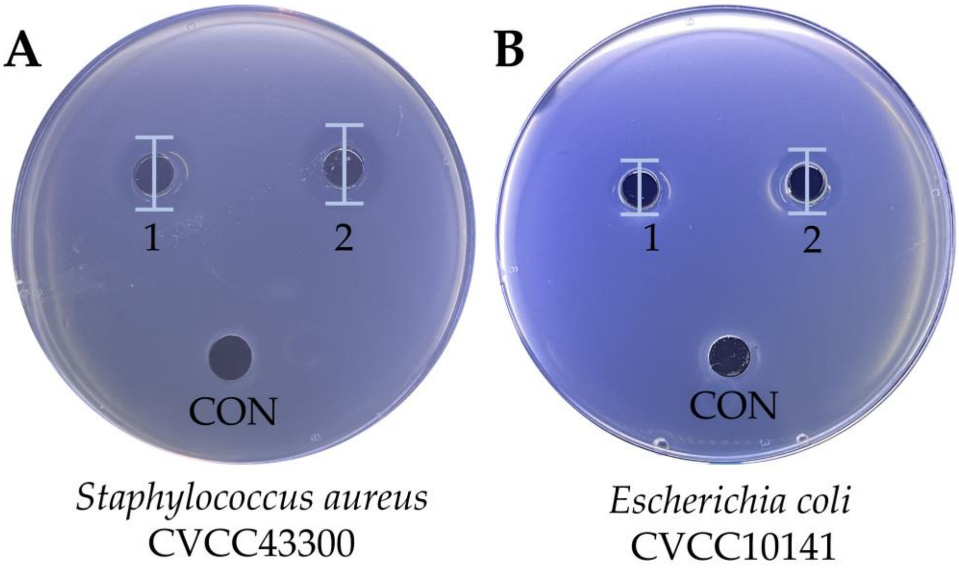

Figure S3. The diameter of inhibition zone of LR-LFCA before and after optimization. (A) *Staphylococcus aureus* CVCC43300. (B) *Escherichia coli* CVCC10141. 1: LR-LFCA supernatant before optimization, 2: LR-LFCA supernatant after optimization.
